# Prognosis after discontinuing renin angiotensin aldosterone system inhibitor for heart failure with restored ejection fraction after acute myocardial infarction

**DOI:** 10.1038/s41598-023-30700-1

**Published:** 2023-03-02

**Authors:** Seung Hun Lee, Tae-Min Rhee, Doosup Shin, David Hong, Ki Hong Choi, Hyun Kuk Kim, Taek Kyu Park, Jeong Hoon Yang, Young Bin Song, Joo-Yong Hahn, Seung-Hyuck Choi, Shung Chull Chae, Myeong-Chan Cho, Chong Jin Kim, Ju Han Kim, Hyo-Soo Kim, Hyeon-Cheol Gwon, Myung Ho Jeong, Joo Myung Lee, The KAMIR-NIH Investigators, Seung Hun Lee, Tae-Min Rhee, Doosup Shin, David Hong, Ki Hong Choi, Hyun Kuk Kim, Taek Kyu Park, Jeong Hoon Yang, Young Bin Song, Joo-Yong Hahn, Seung-Hyuck Choi, Shung Chull Chae, Myeong-Chan Cho, Chong Jin Kim, Ju Han Kim, Hyo-Soo Kim, Hyeon-Cheol Gwon, Myung Ho Jeong, Joo Myung Lee

**Affiliations:** 1 Division of Cardiology, Department of Internal Medicine, Heart Center, Chonnam National University Hospital, Chonnam National University Medical School, Gwangju, Korea; 2 Department of Internal Medicine and Cardiovascular Center, Seoul National University Hospital, Seoul, Korea; 3 Division of Cardiology, Department of Internal Medicine, Duke University Medical Center, Durham, NC USA; 4 Division of Cardiology, Department of Internal Medicine and Cardiovascular Center, Heart Vascular Stroke Institute, Samsung Medical Center, Sungkyunkwan University School of Medicine, 50, Irwon-Dong, Gangnam-Gu, Seoul, 135-710 Korea; 5 Department of Internal Medicine and Cardiovascular Center, Chosun University Hospital, University of Chosun College of Medicine, Gwangju, Korea; 6 Department of Internal Medicine, Kyungpook National University Hospital, Daegu, Korea; 7 Cardiology Division, Department of Internal Medicine, Chungbuk National University Hospital, Cheongju, Korea; 8 Department of Internal Medicine, Kyunghee University College of Medicine, Seoul, Korea

**Keywords:** Cardiology, Cardiovascular biology, Interventional cardiology

## Abstract

Prognostic effect of discontinuing renin–angiotensin–aldosterone-system-inhibitor (RAASi) for patients with heart failure (HF) after acute myocardial infarction (AMI) whose left ventricular (LV) systolic function was restored during follow-up is unknown. To investigate the outcome after discontinuing RAASi in post-AMI HF patients with restored LV ejection fraction (EF). Of 13,104 consecutive patients from the nationwide, multicenter, and prospective Korea Acute Myocardial Infarction-National Institutes of Health (KAMIR-NIH) registry, HF patients with baseline LVEF < 50% that was restored to ≥ 50% at 12-month follow-up were selected. Primary outcome was a composite of all-cause death, spontaneous MI, or rehospitalization for HF at 36-month after index procedure. Of 726 post-AMI HF patients with restored LVEF, 544 maintained RAASi (Maintain-RAASi) beyond 12-month, 108 stopped RAASi (Stop-RAASi), and 74 did not use RAASi (RAASi-Not-Used) at baseline and follow-up. Systemic hemodynamics and cardiac workloads were similar among groups at baseline and during follow-up. Stop-RAASi group showed elevated NT-proBNP than Maintain-RAASi group at 36-month. Stop-RAASi group showed significantly higher risk of primary outcome than Maintain-RAASi group (11.4% vs. 5.4%; adjusted hazard ratio [HR_adjust_] 2.20, 95% confidence interval [CI] 1.09–4.46,* P* = 0.028), mainly driven by increased risk of all-cause death. The rate of primary outcome was similar between Stop-RAASi and RAASi-Not-Used group (11.4% vs. 12.1%; HR_adjust_ 1.18 [0.47–2.99], *P* = 0.725). In post-AMI HF patients with restored LV systolic function, RAASi discontinuation was associated with significantly increased risk of all-cause death, MI, or rehospitalization for HF. Maintaining RAASi will be necessary for post-AMI HF patients, even after LVEF is restored.

## Introduction

Renin–angiotensin–aldosterone-system-inhibitors (RAASi) are recommended as a class I indication for patients with heart failure with reduced ejection fraction (HFrEF) as part of guideline-directed medical therapy (GDMT), as they alleviate symptoms, reduce hospitalizations due to heart failure (HF), and enhance overall survival^1–4^. GDMT for HFrEF can improve myocardial function in up to half of patients^5–7^, and patients with restored myocardial function (heart failure with restored ejection fraction [HFresEF]) tend to have better clinical outcomes^8–11^.

In clinical practice, a common question among patients with HFresEF is whether RAASi should be continued even after recovery of left ventricular (LV) systolic function despite potential issues with adverse drug reaction, polypharmacy, and financial burden^12^. Although the net benefit of continued RAASi for HFresEF has not yet been clearly determined^12^, there is also limited evidence or consensus on the feasibility and safety of discontinuation of RAASi after recovery of LV systolic function^1,13,14^. Notably, a substantial proportion of patients with HFresEF have remaining cardiac pathologies that increase risk of future adverse events, even with continued treatment^15^. Furthermore, in the recent TRED-HF trial, most asymptomatic dilated cardiomyopathy (DCM) patients with HFresEF eventually relapsed after stopping treatment^12^.

In patients with ischemic heart disease, RAASi may further reduce the risk of future coronary events in addition to their benefits on morbidity and mortality related to HF^1,12^. Furthermore, unlike DCM patients in which underlying pathophysiology cannot be changed, impaired myocardial perfusion in patients with AMI can be treated by revascularization, which would help recovery of myocardial function. Therefore, the prognostic impact of continuation or discontinuation of RAASi after recovery of LV systolic function in post-AMI patients may be different from that in DCM patients. In this regard, we aimed to investigate the prognostic impact of discontinuation of RAASi in post-AMI patients with HFresEF using a large prospective registry.

## Methods

### Study population

Study population of the present study was derived from a nationwide, multicenter, and prospective Korean Acute Myocardial Infarction-National Institutes of Health (KAMIR-NIH) registry. The KAMIR-NIH is a dedicated prospective registry which consecutively enrolled AMI patients at 20 tertiary university hospitals eligible for primary PCI from November 2011 to December 2015 without any exclusion criteria. Detailed study protocols have been published elsewhere^16^.

**Figure 1:**
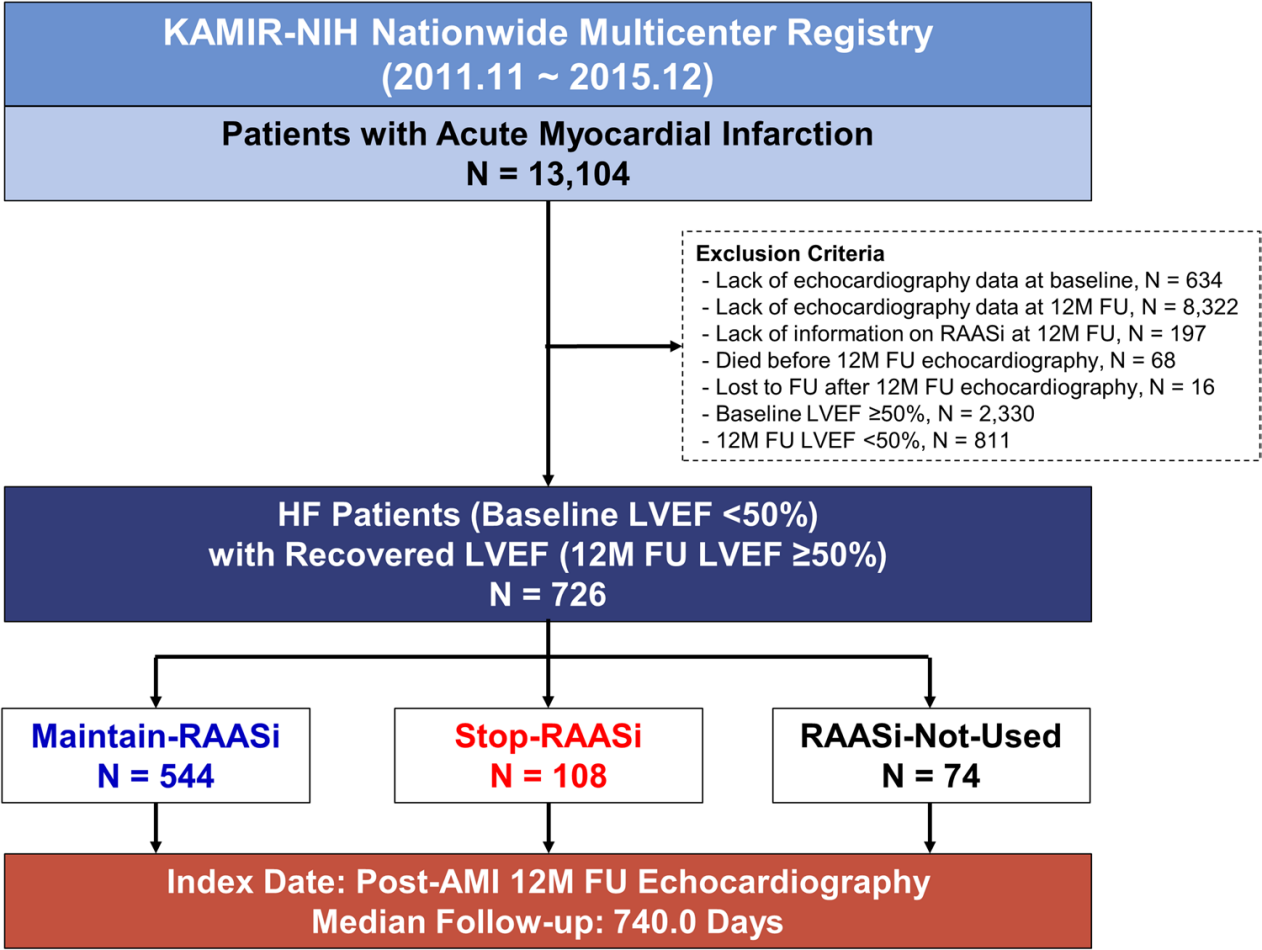
Study flow. Study flow of the present study is shown. From the KAMIR-NIH registry, a total of 726 HFresEF patients were selected for analysis and follow-up during 36-month after index procedure. Abbreviations: AMI, acute myocardial infarction; FU, follow-up; HFresEF, heart failure with restored ejection fraction; KAMIR-NIH, Korea Acute Myocardial Infarction-National Institutes of Health; LVEF, left ventricular ejection fraction; RAASi, renin–angiotensin–aldosterone-system-inhibitor.

For the current analysis, we selected AMI patients with baseline LVEF < 50%, whose LV systolic function restored to ≥ 50% at 12-month follow-up echocardiographic examination. We excluded patients without echocardiography data at baseline or 12-month follow-up, without information on RAASi at 12-month follow-up, who died before 12-month follow-up echocardiography, or who were lost to follow-up after 12-month follow-up echocardiography. Of 13,104 consecutive patients from the KAMIR-NIH registry, 726 patients were selected and classified into 3 groups: (1) those who maintained RAASi treatment both at baseline and 12-month follow-up (Maintain-RAASi group); (2) stopped RAASi at 12-month follow-up, based on restored EF (Stop-RAASi group); or (3) did not use RAASi both at baseline and 12-month follow-up (RAASi-Not-Used group) (Fig. 1). Starting from the index date of 12-month follow-up echocardiography, patients were followed up to 24-month (36-month after AMI).

The protocol of KAMIR-NIH was approved by the ethics committee (the Institutional Review Board of each participating center and Samsung Medical Center, Seoul, Korea) and was conducted according to the principles of the Declaration of Helsinki. All enrolled patients provided written informed consent. In cases of patients being unable to consent due to clinical status, a relative was informed and could provide consent on behalf of that patient.

### Patient management, data collection and follow-up

Patient treatment was performed according to current standard practice. The choice of treatment strategy, type, diameter, and length of stents, the use of medications, intravascular imaging devices, thrombus aspiration, or hemodynamic support devices were left to the operator’s discretion. Unless there was an undisputed reason for discontinuing dual antiplatelet therapy, all patients were recommended to be given aspirin indefinitely plus clopidogrel or other potent antiplatelet agents, such as prasugrel or ticagrelor, for at least 1 year. Choice of P2Y_12_ inhibitors prescribed was left to operator’s discretion in accordance with the guidelines and patient bleeding risk.

Demographic features and cardiovascular risk factors were collected by patient interviews or review of medical records. During hospitalization, findings of coronary angiography and detailed procedural characteristics of percutaneous coronary intervention as well as information on discharge medications were collected. All patients were recommended to perform echocardiography during index admission and during the follow-up period annually after AMI using commercially available ultrasound systems. Assessment of systolic function was performed according to ASE/EACVI recommendations^17^. Follow-up data was recorded during the 36-month of follow-up after discharge. The data was completed by telephone interview if patients did not visit on their scheduled day of follow-up. Using a web-based case report form in the internet-based Clinical Research and Trial management system (iCReaT), independent clinical research coordinators collected all baseline data and clinical events up to 36-month follow-up (iCReaT Study No. C110016).

### Study outcomes

The primary outcome was a composite of all-cause death, spontaneous MI, or rehospitalization for HF. Secondary endpoints included individual components of primary outcome. Spontaneous MI was defined as the recurrence of symptoms or the presence of electrocardiographic changes in association with a rise in cardiac biomarker levels above the upper limit of normal, and periprocedural MI was not included as a clinical outcome. All endpoints were defined according to the Academic Research Consortium definitions^18^. All clinical events were evaluated by an independent event adjudicating committee. The definition of study endpoints and the process of event adjudication is described in the previous publication of KAMIR-NIH investigators^16,19^.

### Statistical analysis

Categorical variables were presented as numbers and relative frequencies (percentages) and were compared using the Chi-squared test. Continuous variables were expressed as mean ± standard deviation and were compared using the analysis of variance (ANOVA). Repeated measure ANOVA test was used to compare the overall difference of repeated measurements of systolic and diastolic blood pressures, heart rate, cardiac workload (calculated by multiplying systolic blood pressure and heart rate), LVEF, and N-terminal pro-B-type natriuretic peptide (NT-proBNP) among the groups. Cumulative incidence of events at 36 months was calculated based on Kaplan–Meier censoring estimates, and comparison of clinical outcomes among groups was performed with the log-rank test. Hazard ratios (HRs) with 95% confidence intervals (CIs) were calculated using univariable and multivariable Cox proportional hazard models. Covariates included in multivariable model were selected if they were significantly different among the 3 groups or considered to have significant predictive values, which were as follows: age, sex, hypertension, diabetes mellitus, and LVEF at baseline. The Cox proportional hazard regression in a propensity-score matched cohort for main comparators (Maintain-RAASi group vs. Stop-RAASi group) was performed. A multivariable logistic regression model was used to generate propensity-scores which indicate the probability that one would be in the Stop-RAASi group. The logistic regression model included age, sex, body mass index, hypertension, diabetes mellitus, dyslipidemia, previous MI, previous angina, previous atrial fibrillation, previous cerebrovascular accident, current smoking, familial history of coronary artery disease, Killip class, presented as STEMI, cardiogenic shock, 3-vessel disease, left main coronary artery disease, use of ECMO, LVEF at baseline, and LVEF at 1-year as covariates. A 1:1 matching process without replacements was performed by a greedy algorithm with a caliper width of 0.25 standard deviations, yielding 105 patients in the Stop-RAASi group matched with 105 controls in the Maintain-RAASi group. All probability values were two-sided and *P* values < 0.05 were considered statistically significant. All analyses were conducted using Stata software, version 14.0 (StataCorp. 2015. Stata Statistical Software: Release 14. College Station, TX: StataCorp LP).

## Results

### Baseline characteristics

Baseline characteristics are described in Table 1. We analyzed 726 AMI patients whose LVEF was below 50% at baseline but restored at 12-month follow-up according to treatment strategy of RAASi (544 maintained RAASi at 12-month [Maintain-RAASi group], 108 stopped RAASi at 12-month, based on restored EF [Stop-RAASi group], and 74 did not use RAASi at baseline or during 12-month follow-up [RAASi-Not-Used group]). Starting from the date of 12-month follow-up echocardiography, median follow-up duration of the study population was 740.0 days. Except for a higher proportion of hypertension in the Maintain-RAASi group, proportions of cardiovascular risk factors were similar among the 3 groups.

**Table 1:** Baseline characteristics according to treatment strategy of RAAS inhibitor.

	Maintain-RAASi (n = 544)	Stop-RAASi (n = 108)	RAASi-Not-Used (n = 74)	*P* value
Demographics				
Age, years	61.7 ± 11.8	63.7 ± 11.8	62.8 ± 11.9	0.26
Male, % (n)	76.7% (417)	73.1% (79)	71.6% (53)	0.52
Cardiovascular Risk factors				
Hypertension, % (n)	45.4% (247)	26.9% (29)	36.5% (27)	0.001
Diabetes mellitus, % (n)	27.8% (151)	23.1% (25)	25.7% (19)	0.60
Dyslipidemia, % (n)	10.7% (58)	9.3% (10)	9.5% (7)	0.88
Previous MI, % (n)	3.7% (20)	6.5% (7)	2.7% (2)	0.33
Previous angina, % (n)	6.6% (36)	12.0% (13)	5.4% (4)	0.11
Previous CHF, % (n)	0.7% (4)	1.9% (2)	0.0% (0)	0.36
Previous AF, % (n)	8.6% (47)	10.2% (11)	12.2% (9)	0.58
Previous CVA, % (n)	5.9% (32)	4.6% (5)	6.8% (5)	0.82
Current smoking, % (n)	45.8% (249)	39.8% (43)	39.2% (29)	0.34
Chronic kidney disease, % (n)	14.2% (77)	17.6% (19)	19.2% (14)	0.40
Familial history of CAD, % (n)	5.0% (27)	3.7% (4)	9.5% (7)	0.20
Initial Presentation				
Killip class, % (n)				0.66
I	76.1% (414)	83.3% (90)	78.4% (58)	
II	12.7% (69)	6.5% (7)	10.8% (8)	
III	6.1% (33)	6.5% (7)	5.4% (4)	
IV	5.1% (28)	3.7% (4)	5.4% (4)	
Presented as STEMI, % (n)	65.1% (354)	62.0% (67)	58.1% (43)	0.46
Cardiogenic shock, % (n)	5.1% (28)	4.6% (5)	12.2% (9)	0.045
Cardiopulmonary resuscitation, % (n)	5.0% (27)	2.8% (3)	10.8% (8)	0.049
Multivessel disease, % (n)	51.7% (281)	41.7% (45)	44.6% (33)	0.11
3-vessel disease, % (n)	16.5% (90)	17.6% (19)	17.6% (13)	0.95
Left main coronary artery disease, % (n)	4.0% (22)	4.6% (5)	6.8% (5)	0.56
Procedural Characteristics				
Non-culprit vessel revascularization during index admission, % (n)	63.8% (347)	69.4% (75)	63.5% (47)	0.52
Use of IABP, % (n)	1.7% (9)	1.9% (2)	1.4% (1)	0.97
Use of ECMO, % (n)	0.2% (1)	0.9% (1)	0.0% (0)	0.36
Implantation of ICD, % (n)	0.2% (1)	0.0% (0)	0.0% (0)	0.85
Discharge Medication				
Aspirin	100.0% (544)	100.0% (108)	100.0% (74)	1.00
Clopidogrel	77.6% (422)	72.2% (78)	70.3% (52)	0.23
Prasugrel	12.1% (66)	13.0% (14)	12.2% (9)	0.97
Ticagrelor	23.0% (125)	22.2% (24)	27.0% (20)	0.71
RAASi				< 0.001
Angiotensin-converting enzyme inhibitor	73.9% (402)	68.5% (74)	0.0% (0)	
Angiotensin receptor blocker	26.1% (142)	31.5% (34)	0.0% (0)	
Beta-blocker	91.5% (498)	91.7% (99)	79.7% (59)	0.005
Statin	95.0% (517)	94.4% (102)	94.6% (70)	0.96
Oral anticoagulant	3.9% (21)	5.6% (6)	6.8% (5)	0.43
Concomitant medications at 1 year				
Aspirin	90.6% (493)	83.3% (90)	83.8% (62)	0.031
Clopidogrel	64.7% (352)	53.7% (58)	60.8% (45)	0.091
Prasugrel	4.2% (23)	5.6% (6)	5.4% (4)	0.78
Ticagrelor	4.4% (24)	0.0% (0)	5.4% (4)	0.072
RAASi				< 0.001
Angiotensin-converting enzyme inhibitor	46.1% (251)	0.0% (0)	0.0% (0)	
Angiotensin receptor blocker	53.9% (293)	0.0% (0)	0.0% (0)	
Beta-blocker	85.3% (464)	77.8% (84)	70.3% (52)	0.002
Statin	96.3% (524)	78.7% (85)	89.2% (66)	< 0.001
Oral anticoagulant	2.2% (12)	1.9% (2)	2.7% (2)	0.93

AF, atrial fibrillation; CAD, coronary artery disease; CHF, congestive heart failure; CVA, cerebrovascular accident; ECMO, extracorporeal membrane oxygenator; IABP, intra-aortic balloon pump; ICD, implantable cardioverter-defibrillator; MI, myocardial infarction; RAASi, renin–angiotensin–aldosterone system inhibitor; STEMI, ST-segment elevation myocardial infarction.

Patients who presented with cardiogenic shock or received cardiopulmonary resuscitation were more prevalent in RAASi-Not-Used group than others. However, procedural characteristics including use of mechanical hemodynamic support were not different among the 3 groups. Except RAASi, profiles of discharge medications were similar among groups. At discharge, 73.9% used angiotensin-converting enzyme inhibitors and 26.1% angiotensin receptor blockers, while the proportion of angiotensin receptor blockers increased to 53.9% at 12-month and angiotensin-converting enzyme inhibitors decreased to 46.1%.

### Change of systemic hemodynamics and biomarkers during follow-up

**Figure 2:**
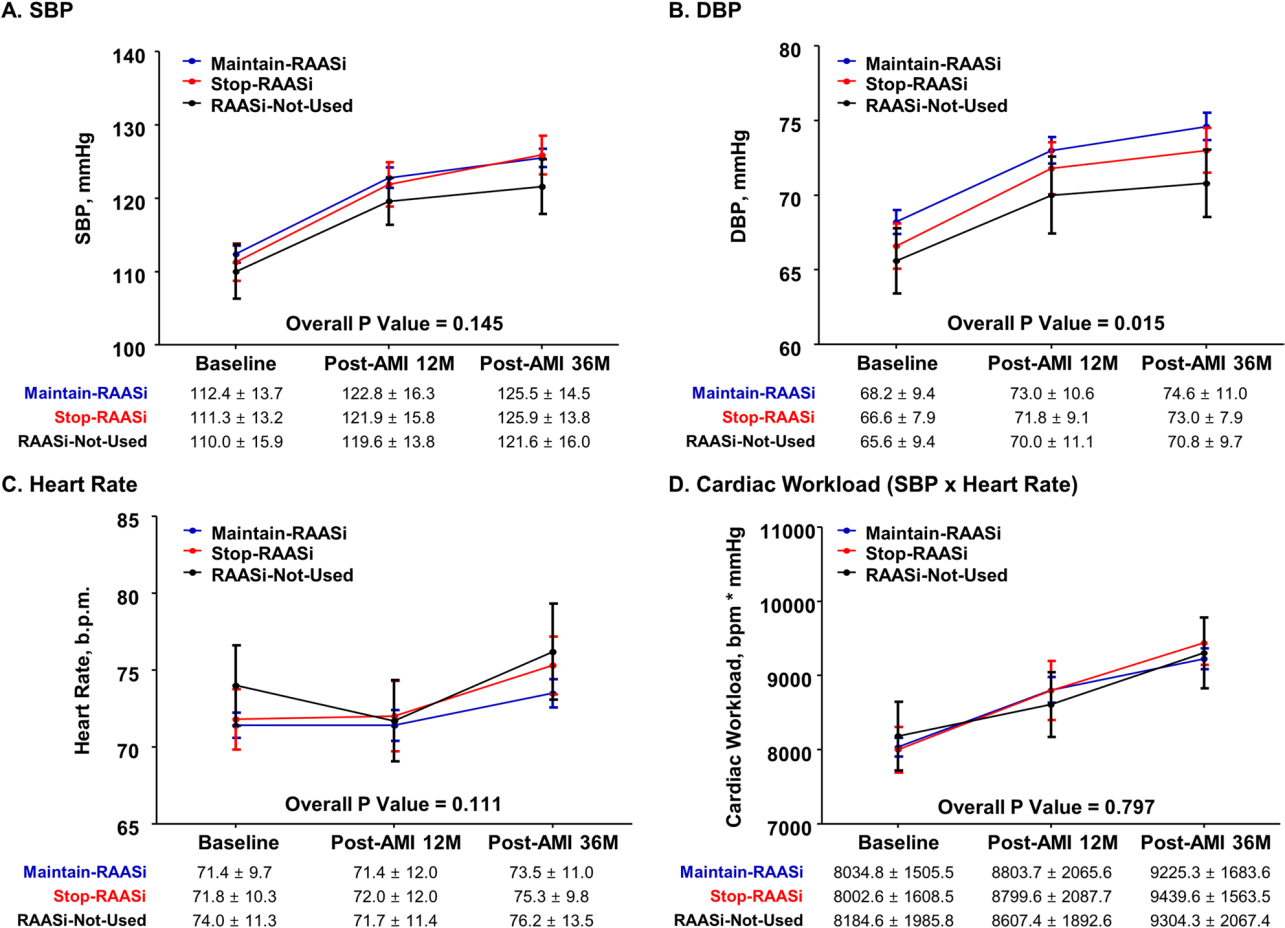
Changes of systemic hemodynamics according to treatment strategy of RAAS inhibitor after post-AMI 12-month. Serial change of systemic hemodynamic parameters, (**A**) SBP, (**B**) DBP, (**C**) heart rate, and (**D**) cardiac workload is presented in 3 groups; Maintain-RAASi, Stop-RAASi, and RAASi-Not-Used groups. Abbreviations: b.p.m., beats per minute; DBP, diastolic blood pressure; SBP, systolic blood pressure; otherwise as in Fig. 1.

Systolic blood pressure, diastolic blood pressure, and cardiac workload consistently increased after the index procedure during the follow-up (Fig. 2). Among the 3 groups, Maintain-RAASi and Stop-RAASi groups showed higher systolic and diastolic blood pressure than RAASi-Not-Used group at baseline, 12-month, and 36-month follow-up. Conversely, cardiac workload was similar among the 3 groups at baseline, 12-month, and 36-month follow-up.

**Figure 3:**
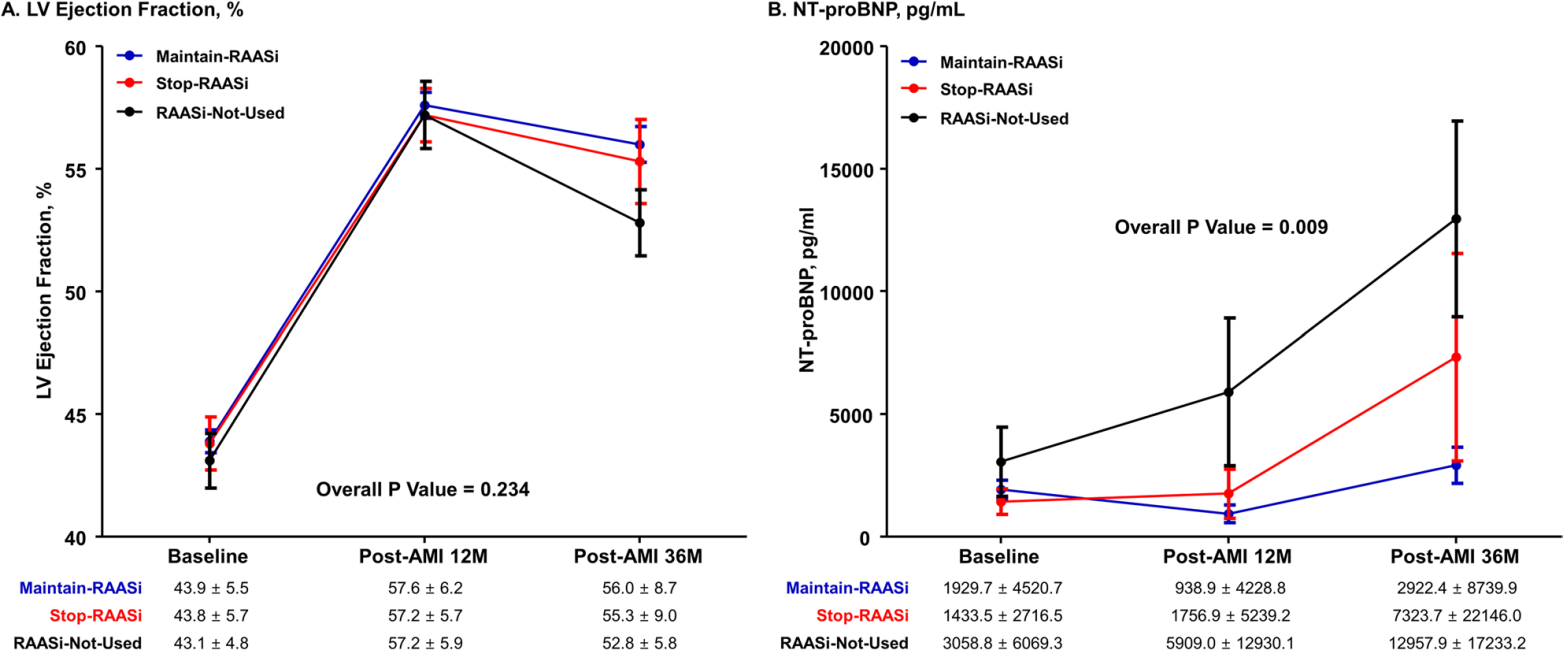
Changes of LV systolic function and levels of NT-proBNP according to Treatment Strategy of RAAS Inhibitor after Post-AMI 12-Month. Serial change of (**A**) LV ejection fraction, and (**B**) concentration of NT-proBNP is presented in 3 groups; Maintain-RAASi, Stop-RAASi, and RAASi-Not-Used groups. Abbreviations: LV, left ventricular; NT-proBNP, N-terminal pro-B-type natriuretic peptide; otherwise as in Fig. 1.

LVEF at 12-months restored similarly among all groups (Maintain-RAASi group, 57.6 ± 6.2%; Stop-RAASi group, 57.2 ± 5.7%; RAASi-Not-Used group, 57.2 ± 5.9%), and tended to decrease more in RAASi-Not-Used group (52.8 ± 5.8%) than Stop-RAASi group (55.3 ± 9.0%) and Maintain-RAASi group (56.0 ± 8.7%) at 36-month (Fig. 3). The NT-proBNP level was similar among the 3 groups at baseline, however, Stop-RAASi group showed increased NT-proBNP level than Maintain-RAASi group at both 12-month and 36-month. RAASi-Not-Used group showed the highest level of NT-proBNP during the follow-up period (Fig. 3).

### Outcomes at 36-month according to treatment strategy of RAAS inhibitor

**Figure 4:**
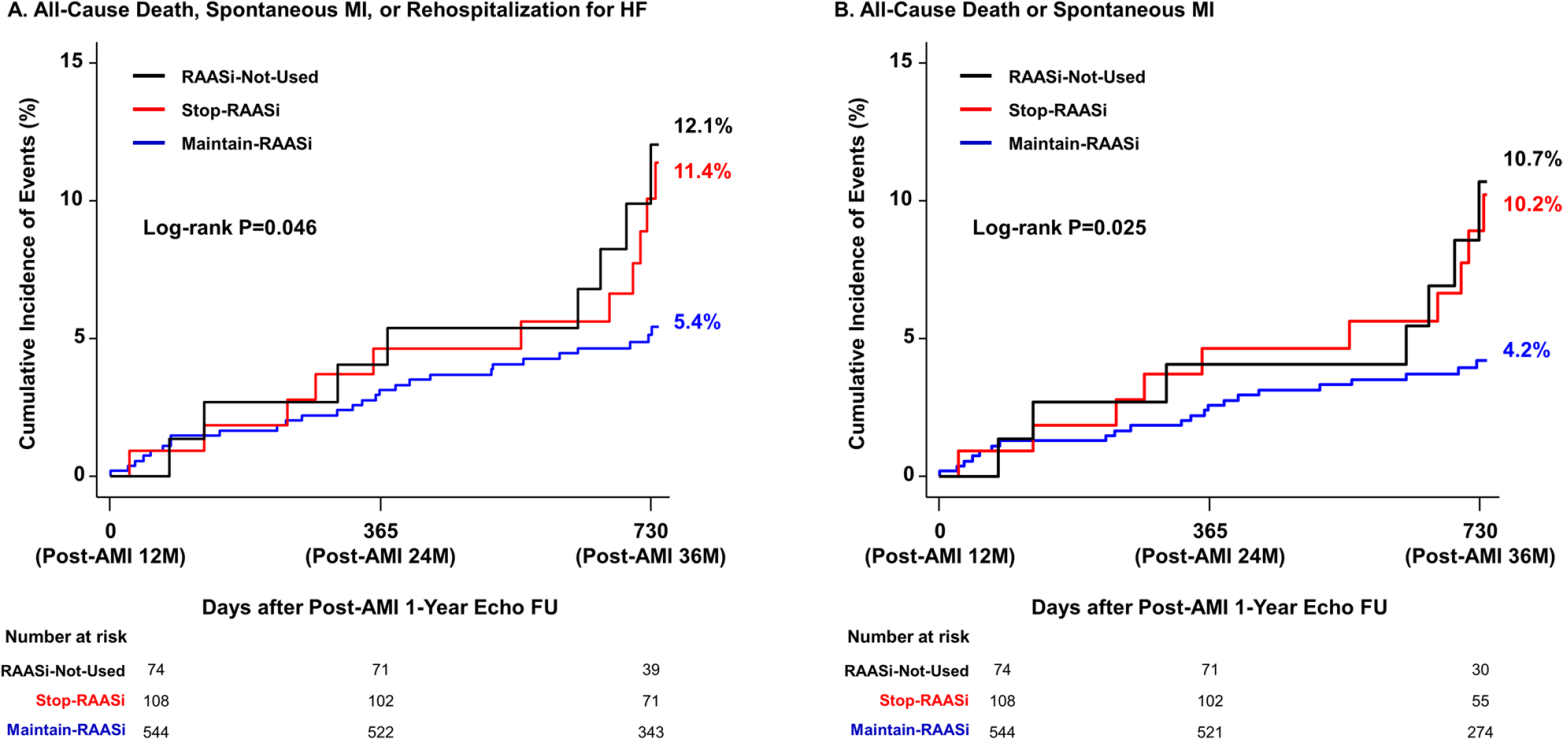
Comparison of composite of all-cause death or spontaneous MI after post-AMI 12-month according to treatment strategy of RAAS inhibitor. Comparison of cumulative incidence and Kaplan–Meier curves of (**A**) a composite of all-cause death, spontaneous MI, or rehospitalization for HF, and (**B**) all-cause death or spontaneous MI among the 3 groups; Maintain-RAASi, Stop-RAASi, and RAASi-Not-Used groups, are presented. Abbreviations: HF, heart failure, otherwise as in Fig. 1.

At 36-month after index procedure, the risk of all-cause death, spontaneous MI, or rehospitalization due to HF in Stop-RAASi group was significantly higher than Maintain-RAASi group (11.4% vs. 5.4%; adjusted HR 2.09, 95% CI 1.02–4.28, P = 0.043), mainly driven by increased risk of all-cause death in the Stop-RAASi group. Regarding the main comparator groups (Stop-RAASi group vs. Maintain-RAASi group), the proportionality assumptions were not violated for all outcomes (Supplementary Fig. 1 and 2). The cumulative incidence of primary outcome was similar between Stop-RAASi group and RAASi-Not-Used group (11.4% vs. 12.1%; adjusted HR 1.18, 95% CI 0.47–2.99, *P* = 0.725) (Table 2 and Fig. 4). Comparison of secondary outcomes showed similar trends (Supplementary Fig. 3). However, outcomes related with coronary revascularization, especially target vessel or target lesion revascularization, were similar among three groups (Supplementary Table 4). The results were consistently observed in a well-balanced 1:1 propensity-score matched cohort (Supplementary Table 1 and 2). The results were also consistent in another multivariable model including aspirin, beta-blocker, and statin use at 1-year (Supplementary Table 3).

**Table 2:** Comparison of clinical outcomes after 1-year echocardiography follow-up according to treatment strategy of RAAS inhibitor.

Events	Cumulative Incidence of Events (%)*			Risk of Events in Group 2 (Group 1 as reference)		Risk of Events in Group 2 (Group 3 as reference)	
	Group 1 (Maintain-RAASi)	Group 2 (Stop-RAASi)	Group 3 (RAASi-Not-Used)	Adjusted HR (95% CI)^†^	P value	Adjusted HR (95% CI)^†^	*P* value
All-cause death, spontaneous MI or Rehospitalization due to HF	5.4% (28)	11.4% (11)	12.1% (8)	2.09 (1.02–4.28)	0.043	1.18 (0.47–2.99)	0.725
All-cause death or spontaneous MI	4.2% (22)	10.2% (10)	10.7% (7)	2.43 (1.12–5.23)	0.024	1.24 (0.46–3.33)	0.665
All-cause death	2.3% (12)	7.0% (7)	7.9% (5)	2.88 (1.10–7.56)	0.031	1.17 (0.36–3.80)	0.788
Spontaneous MI	2.1% (11)	3.5% (3)	2.9% (2)	1.72 (0.46–6.37)	0.420	1.24 (0.20–7.57)	0.818
Rehospitalization for HF	2.8% (14)	5.5% (5)	5.1% (3)	1.92 (0.68–5.45)	0.219	1.39 (0.33–5.91)	0.655

CI, confidence interval; HF, heart failure; HR, hazard ratio; MI, myocardial infarction; RAASi, renin–angiotensin–aldosterone system inhibitor.

*The cumulative incidence of clinical outcomes is presented as Kaplan–Meier estimates at 3-year from index procedure.

^**†**^Multivariable Cox regression model included age, sex, hypertension, diabetes mellitus, and left ventricular ejection fraction at baseline as covariates.

## Discussion

In this nationwide, multicenter, and prospective AMI registry, we evaluated the prognostic impact of discontinuation of RAASi in HFresEF patients. Major findings were as follows: (1) Although LVEF at 36 months was similar between the Maintain-RAASi group and the Stop-RAASi group, NT-proBNP level at 36 months was the lowest in the Maintain-RAASi group among the 3 groups; (2) the Stop-RAASi group had a significantly higher cumulative incidence of all-cause death, MI, or HF readmission compared with the Maintain-RAASi group; and (3) cumulative incidence of all-cause death, MI, or HF readmission in the Stop-RAASi group was similar with that of RAASi-Not-Used group.

### Discontinuation of GDMT after recovery of LV systolic function in HF patients

GDMT for HF can improve LVEF by > 10% in more than one-third of patients with HF^20,21^. Reverse remodeling of the LV, which is defined by decrease in LV size, regression of hypertrophy and fibrosis, and improvement in systolic function, is well known to be associated with improved symptoms and clinical outcomes^20,22^. In this regard, recent guidelines developed a new subset of HFrEF patients whose LVEF is restored (HFresEF)^23,24^. The need for continuation of HF medications after recovery of LVEF can sometimes be questioned due to adverse drug reactions, financial burden, polypharmacy, and uncertain benefit of indefinite HF medications in patients with HFresEF^25^.

To date, a few anecdotal studies and one randomized trial investigated the impact of discontinuation of medications in various subgroups of HF patients^25–29^. Discontinuation of HF medications after recovery of LVEF in DCM patients was associated with relapse of HF and LV dysfunction^25,29^. In a small study with 15 patients with HFresEF, discontinuing beta-blockers resulted in a substantial decrease in LVEF, which was then restored after resumption of the beta-blockers^27^. Recently, the TRED-HF trial exclusively evaluated DCM patients and reported that approximately half of patients in the medication discontinuation group eventually had a substantial decline in LV systolic function, a rise in LV end-diastolic volume or NT-proBNP level, or clinical deterioration^25^. Furthermore, in the substudy of TRED-HF trial, medication discontinuation resulted in rapid remodeling of LV, with early tissue and functional changes, even among patients who did not relapse^30^. However, these studies were primarily focused on patients with DCM, and more importantly, could not investigate the impact of medication discontinuation on mortality.

### Continuation of GDMT in Post-AMI Patients with HFresEF

In contrast to DCM patients for whom GDMT is almost their only therapeutic option, successful revascularization in post-AMI patients with HFrEF can help recovery of viable myocardium and LV systolic function. Therefore, the prognostic impact of discontinuation of HF medications after recovery of LVEF can be different between the 2 patient populations. However, there has been no previous study focusing on post-AMI patients with HFresEF. In the current study, discontinuation of RAASi was associated with significantly higher NT-proBNP levels and cumulative incidence of all-cause death, MI, or HF readmission compared with continuation of RAASi in post-AMI patients with HFresEF. Importantly, discontinuation of RAASi was associated with almost 3 times higher risk of death than continuation of RAASi in this patient population. Interestingly, patient prognosis in the Stop-RAASi group was comparable with that in the RAASi-Not-Used group.

Our findings corroborate prior studies from DCM patients demonstrating that the recovery of systolic function per se should not be taken as a cure of HF but rather as a remission or temporary discontinuation of a negative stimulus^31^, providing a rationale for indefinite GDMT without interruption in patients with HFrEF due to their ischemic etiology even after successful revascularization^32^. Notably, the present study was particularly important since it first demonstrated (1) impact of treatment discontinuation in the homogenous population of ischemic HF occurred after AMI and (2) increased mortality after discontinuation of HF medications in patients with HFresEF. Another issue regarding the prognostic impact of RAASi in patients with ischemic HF is reduction in additional coronary events, such as spontaneous MI^25^. In the current results, the 2-year risk of spontaneous MI was numerically higher in the Stop-RAASi group than the Maintain-RAASi group without statistical significance (3.5% vs. 2.1%, respectively). Larger prospective studies with longer follow-up duration are required to clarify whether discontinuation HF medications, especially RAASi, would increase future coronary events.

### Possible mechanisms and future perspectives

In general, relapsing HF and related events in patients with HFresEF might be caused by the partial reversal of the HF phenotypes superimposed on the permanent myocardial damage^33^. It is known that even with continued GDMT, many HFresEF patients have remaining impaired cardiac mechanics and are still at significant risk for clinical events^34^. Recent TRED-HF substudy presented that short-term unfavorable remodeling after discontinuation of RAASi might be facilitated by cellular alterations such as calcium dysregulation, energetic malfunction, or sarcomeric dysfunction^30^. In the present study, although LVEF was similar between the Maintain-RAASi group and the Stop-RAASi group (56.0 ± 8.7% vs. 55.3 ± 9.0%, respectively), there was substantial difference in NT-proBNP levels between the 2 groups at 24-month follow-up (36 months after AMI), which suggested unfavorable cardiac mechanics and remodeling with discontinuation of RAASi. Although our data did not include any parameters of cardiac structure or function other than LVEF, these results strongly suggest that recovered LV function alone is not a clinically useful indicator that may advise discontinuation of HF medications, even in post-AMI HFresEF patients^32^. Also, these observations implied the importance of regular surveillance and continuation of GDMT including RAASi in HFresEF patients^35^. Future studies should determine which component of GDMT is more critical to prevent relapse, and also should focus on elucidating the characteristics that distinguish remission from permanent recovery in post-AMI HFresEF patients. In addition, further analysis to evaluate the change of cardiac structural and functional parameters and the subclinical markers of LV systolic function such as LV strain after discontinuing RAASi in HFresEF.

### Study limitations

Several limitations should be discussed. First, since we analyzed an observational prospective cohort, unmeasured confounding factors may have altered the results. Furthermore, the limited sample size of the Stop-RAASi groups and borderline statistical significance in the propensity score matched group analysis should be interpreted with caution. Until future randomized controlled trial is available, the current study results should be interpreted as hypothesis-generating study. Second, selection bias cannot be excluded since a substantial number of participants were omitted due to a lack of echocardiography data at post-AMI 12-month follow-up. Third, the number of analyzed patients in this study might not be sufficient to identify significant differences in LVEF or NT-proBNP level among the 3 groups. Fourth, the reason for discontinuing RAASi of each patient was not clearly reported. Finally, the effect of different proportions of patients who were taking beta-blockers or statins at 12-month after AMI between the Maintain-RAASi and the Stop-RAASi groups cannot be ignored.

## Conclusion

In post-AMI HF patients with restored LV systolic function, discontinuation of RAASi was associated with a significantly increased risk of all-cause death, MI, or rehospitalization for HF. This result strongly suggests the importance of continuation of RAASi in post-AMI patients with HFrEF, even after recovery of LVEF. Further randomized controlled trial is needed to confirm the results from the current hypothesis-generating study.

## Supplementary Information

Supplementary Information.

## Data Availability

The datasets generated and/or analysed during the current study are not publicly available due to the data management policy of the KAMIR investigators, but are available from the corresponding author on reasonable request.

## Abbreviations

AMI: Acute myocardial infarction
CI: Confidence interval
DCM: Dilated cardiomyopathy
EF: Ejection fraction
HFresEF: Heart failure with restored ejection fraction
HR: Hazard ratio
MI: Myocardial infarction
NT-proBNP: N-terminal pro-B-type natriuretic peptide
PCI: Percutaneous coronary intervention
RAASi: Renin–angiotensin–aldosterone-system-inhibitors
