# Supplementary material for: Prognosis after discontinuing renin angiotensin aldosterone system inhibitor for heart failure with restored ejection fraction after acute myocardial infarction
Source: Sci Rep. 2023 Mar 2;13:3539. doi: 10.1038/s41598-023-30700-1 (PMC9981744; doi:10.1038/s41598-023-30700-1)
Supplement: Supplementary file 1 — Supplementary Information. [file 41598_2023_30700_MOESM1_ESM.docx]

**Supplementary Appendix**

**Prognosis after Discontinuing Renin Angiotensin Aldosterone System Inhibitor for Heart Failure with Restored Ejection Fraction after Acute Myocardial Infarction**

Seung Hun Lee, MD, PhD^1†^, Tae-Min Rhee, MD^2†^, Doosup Shin, MD^3^, David Hong, MD^4^, Ki Hong Choi, MD, PhD^4^, Hyun Kuk Kim, MD, PhD^5^, Taek Kyu Park, MD^4^, Jeong Hoon Yang, MD, PhD^4^, Young Bin Song, MD, PhD^4^, Joo-Yong Hahn, MD, PhD^4^, Seung-Hyuck Choi, MD, PhD^4^, Shung Chull Chae, MD, PhD^6^, Myeong-Chan Cho, MD, PhD^7^, Chong Jin Kim, MD, PhD^8^, Ju Han Kim, MD, PhD^1^, Hyo-Soo Kim, MD, PhD^2^, Hyeon-Cheol Gwon, MD, PhD^4^, Myung Ho Jeong, MD, PhD^1^, Joo Myung Lee, MD, MPH, PhD^4^, The KAMIR Investigators

**Table of Contents**

1. **Supplementary Tables**
2. **Supplementary Figures**

**1. Supplementary Tables**

**Supplementary Table 1. Baseline characteristics of propensity-score matched population according to treatment strategy of RAAS inhibitor**

|  | **Maintain-RAASi**  **(n=105)** | **Stop-RAASi**  **(n=105)** | **P value** |
| --- | --- | --- | --- |
| Age, years | 65.2 ± 11.2 | 64.0 ± 11.8 | 0.47 |
| Male, % (n) | 72.4% (76) | 73.3% (77) | 0.88 |
| Body mass index, kg/m^2^ | 23.3 ± 3.0 | 23.4 ± 3.1 | 0.79 |
| Hypertension, % (n) | 27.6% (29) | 26.7% (28) | 0.88 |
| Diabetes mellitus, % (n) | 27.6% (29) | 21.9% (23) | 0.34 |
| Dyslipidemia, % (n) | 11.4% (12) | 9.5% (10) | 0.65 |
| Previous MI, % (n) | 7.6% (8) | 5.7% (6) | 0.58 |
| Previous angina, % (n) | 12.4% (13) | 11.4% (12) | 0.83 |
| Previous CHF, % (n) | 2.9% (3) | 1.9% (2) | 0.65 |
| Previous AF, % (n) | 6.7% (7) | 9.5% (10) | 0.45 |
| Previous CVA, % (n) | 4.8% (5) | 4.8% (5) | 1.00 |
| Current smoking, % (n) | 34.3% (36) | 40.0% (42) | 0.39 |
| Chronic kidney disease, % (n) | 19.0% (20) | 17.1% (18) | 0.72 |
| Familial history of CAD, % (n) | 4.8% (5) | 2.9% (3) | 0.47 |
| Killip class, % (n) |  |  | 0.10 |
| I | 81.0% (85) | 84.8% (89) |  |
| II | 13.3% (14) | 5.7% (6) |  |
| III | 1.9% (2) | 6.7% (7) |  |
| IV | 3.8% (4) | 2.9% (3) |  |
| Presented as STEMI, % (n) | 63.8% (67) | 62.9% (66) | 0.89 |
| Cardiogenic shock, % (n) | 2.9% (3) | 4.8% (5) | 0.47 |
| 3-vessel disease, % (n) | 18.1% (19) | 17.1% (18) | 0.86 |
| Left main coronary artery disease, % (n) | 4.8% (5) | 4.8% (5) | 1.00 |
| Use of ECMO, % (n) | 1.0% (1) | 1.0% (1) | 1.00 |
| LVEF at baseline, % | 44.2 ± 4.8 | 43.7 ± 5.7 | 0.50 |
| LVEF at 1-year, % | 57.5 ± 6.2 | 57.2 ± 5.8 | 0.69 |

Abbreviations: AF, atrial fibrillation; ASD, absolute standardized difference; CAD, coronary artery disease; CHF, congestive heart failure; CVA, cerebrovascular accident; ECMO, extracorporeal membrane oxygenator; LVEF, left ventricular ejection fraction; MI, myocardial infarction; RAASi, renin-angiotensin-aldosterone system inhibitor; STEMI, ST-segment elevation myocardial infarction.

**Supplementary Table 2. Comparison of clinical outcomes after 1-year echocardiography follow-up in propensity-score matched population**

|  | **Cumulative Incidence of Events (%)^*^** | | **Risk of Events in Group 2 (Group 1 as reference)** | | |
| --- | --- | --- | --- | --- | --- |
|  | **Group 1**  **(Maintain-RAASi)**  **(n=105)** | **Group 2**  **(Stop-RAASi)**  **(n=105)** | **PS-matched HR (95% CI)^†^** | **Cox-PH P value** | **Log-rank P value** |
| **All-cause death, spontaneous MI or Rehospitalization due to HF** | 3.4% (3) | 10.4% (10) | 3.38 (0.93-12.29) | 0.064 | 0.049 |
| **All-cause death or spontaneous MI** | 3.4% (3) | 9.2% (9) | 3.04 (0.82-11.25) | 0.095 | 0.079 |
| **All-cause death** | 1.3% (1) | 7.1% (7) | 7.08 (0.87-57.51) | 0.067 | 0.033 |
| **Spontaneous MI** | 2.1% (2) | 2.1% (2) | 1.02 (0.14-7.23) | 0.985 | 0.985 |
| **Rehospitalization for HF** | 1.3% (1) | 5.7% (5) | 5.04 (0.59-43.14) | 0.140 | 0.101 |

**^*^**The cumulative incidence of clinical outcomes is presented as Kaplan-Meier estimates at 3-year from index procedure.

**^†^** The logistic regression model to calculate propensity score included age, sex, body mass index, hypertension, diabetes mellitus, dyslipidemia, previous MI, previous angina, previous AF, previous CVA, current smoking, familial history of CAD, Killip class, presented as STEMI, cardiogenic shock, 3-vessel disease, left main coronary artery disease, use of ECMO, LVEF at baseline, and LVEF at 1-year as covariates.

Abbreviations: AF, atrial fibrillation; ASD, absolute standardized difference; CAD, coronary artery disease; CHF, congestive heart failure; CI, confidence interval; Cox-PH, Cox proportional hazards; CVA, cerebrovascular accident; ECMO, extracorporeal membrane oxygenator; HR, hazard ratio; LVEF, left ventricular ejection fraction; MI, myocardial infarction; RAASi, renin-angiotensin-aldosterone system inhibitor; STEMI, ST-segment elevation myocardial infarction.

**Supplementary Table 3. Sensitivity analysis for the comparison of clinical outcomes after 1-year echocardiography follow-up according to treatment strategy of RAAS inhibitor with different multivariable model**

| **Events** | **Cumulative Incidence of Events (%)^*^** | | | **Risk of Events in Group 2 (Group 1 as reference)** | | **Risk of Events in Group 2 (Group 3 as reference)** | |
| --- | --- | --- | --- | --- | --- | --- | --- |
|  | **Group 1**  **(Maintain-RAASi)** | **Group 2**  **(Stop-RAASi)** | **Group 3**  **(RAASi-Not-Used)** | **Adjusted HR (95% CI)^†^** | **P value** | **Adjusted HR (95% CI)^†^** | **P value** |
| **All-cause death, spontaneous MI or Rehospitalization due to HF** | 5.4% (28) | 11.4% (11) | 12.1% (8) | 2.03 (0.88-4.69) | 0.097 | 1.08 (0.40-2.95) | 0.877 |
| **All-cause death or spontaneous MI** | 4.2% (22) | 10.2% (10) | 10.7% (7) | 2.38 (0.98-5.78) | 0.057 | 1.27 (0.43-3.70) | 0.669 |
| **All-cause death** | 2.3% (12) | 7.0% (7) | 7.9% (5) | 3.01 (1.01-9.01) | 0.048 | 1.25 (0.35-4.46) | 0.726 |
| **Spontaneous MI** | 2.1% (11) | 3.5% (3) | 2.9% (2) | 1.44 (0.28-7.28) | 0.661 | 1.23 (0.15-9.96) | 0.850 |
| **Rehospitalization for HF** | 2.8% (14) | 5.5% (5) | 5.1% (3) | 2.68 (0.83-8.67) | 0.101 | 1.60 (0.35-7.41) | 0.547 |

**^*^**The cumulative incidence of clinical outcomes is presented as Kaplan-Meier estimates at 3-year from index procedure.

**^†^**Multivariable Cox regression model included sex, BMI, Killip class, HTN, DM, previous CVA, LM disease, LVEDD at baseline, BB at discharge, aspirin at 1-year, BB at 1-year, statin at 1-year as covariates.

Abbreviations: BB, beta-blocker; BMI, body mass index; CI, confidence interval; CVA, cerebrovascular accident; DM, diabetes mellitus; HF, heart failure; HR, hazard ratio; HTN, hypertension; LM, left main; LVEDD, left ventricular end-diastolic dimension; MI, myocardial infarction; RAASi, renin-angiotensin-aldosterone system inhibitor.

**Supplementary Table 4. Coronary revascularization after 1-year echocardiography follow-up according to treatment strategy of RAAS inhibitor**

| **Events** | **Cumulative Incidence of Events (%)^*^** | | |
| --- | --- | --- | --- |
|  | **Group 1**  **(Maintain-RAASi)** | **Group 2**  **(Stop-RAASi)** | **Group 3**  **(RAASi-Not-Used)** |
| **Any repeat revascularization** | 6.4% (34) | 4.0% (4) | 2.8% (2) |
| **Target vessel revascularization** | 4.4% (22) | 4.1% (4) | 2.8% (2) |
| **Target lesion revascularization** | 2.0% (9) | 3.0% (3) | 1.4% (1) |
| **Coronary artery bypass graft surgery** | 0.0% (0) | 0.0% (0) | 1.4% (1) |

**^*^**The cumulative incidence of clinical outcomes is presented as Kaplan-Meier estimates at 3-year from index procedure.

Abbreviations: RAASi, renin-angiotensin-aldosterone system inhibitor.

**Supplementary Figure 1. Log-minus-log Plot for Composite Endpoints after Post-AMI 12-Months According to Treatment Strategy of RAAS Inhibitor**
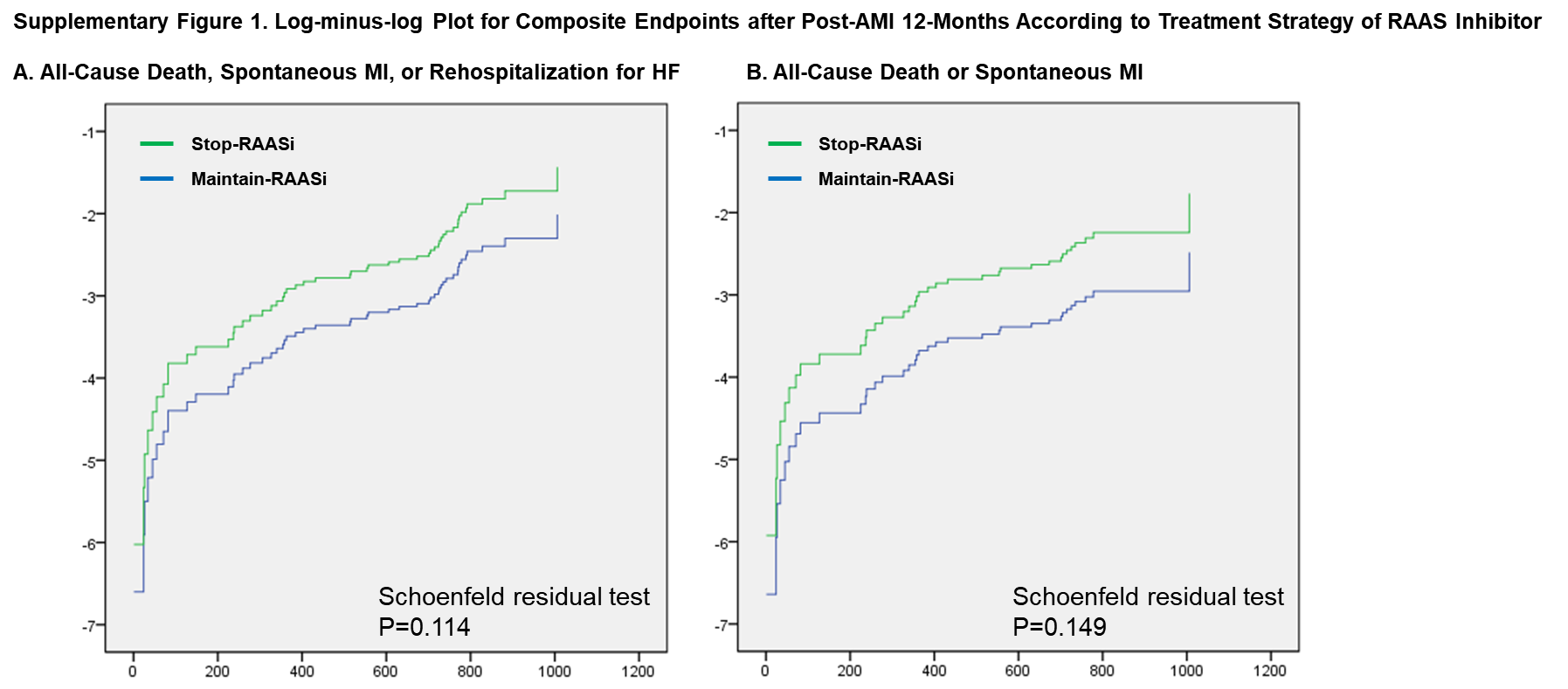


Log-minus-log curves with results of Schoenfeld residual test are shown for (A) a composite of all-cause death, spontaneous MI, or rehospitalization for HF, and (B) all-cause death or spontaneous MI in the Stop-RAASi and Maintain-RAASi groups.

Abbreviations: AMI, acute myocardial infarction; HF, heart failure; MI, myocardial infarction; RAASi, Renin-Angiotensin-Aldosterone System inhibitor.

**Supplementary Figure 2. Log-minus-log Plot for Individual Outcomes after Post-AMI 12-Months According to Treatment Strategy of RAAS Inhibitor**


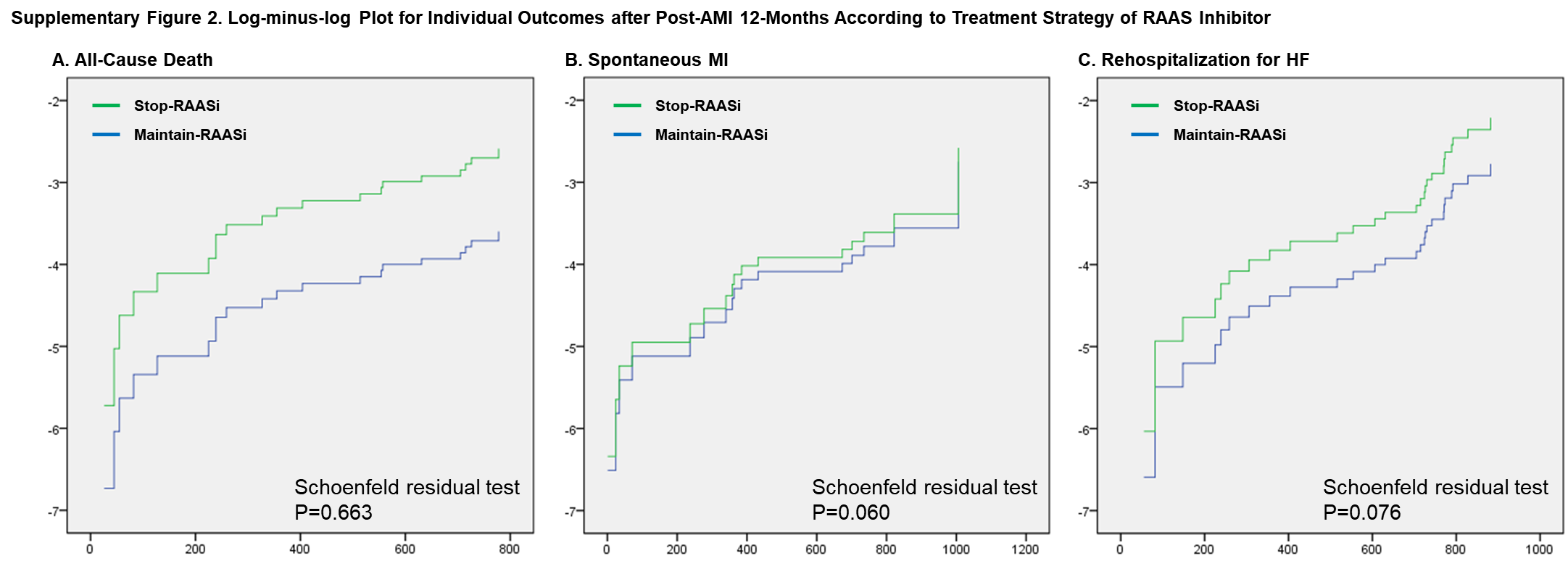


Log-minus-log curves with results of Schoenfeld residual test are shown for (A) all-cause death, (B) spontaneous MI, and (C) rehospitalization for HF in the Stop-RAASi and Maintain-RAASi groups.

Abbreviations: AMI, acute myocardial infarction; HF, heart failure; MI, myocardial infarction; RAASi, Renin-Angiotensin-Aldosterone System inhibitor.

**Supplementary Figure 3. Comparison of Individual Outcomes after Post-AMI 12-Months According to Treatment Strategy of RAAS Inhibitor**


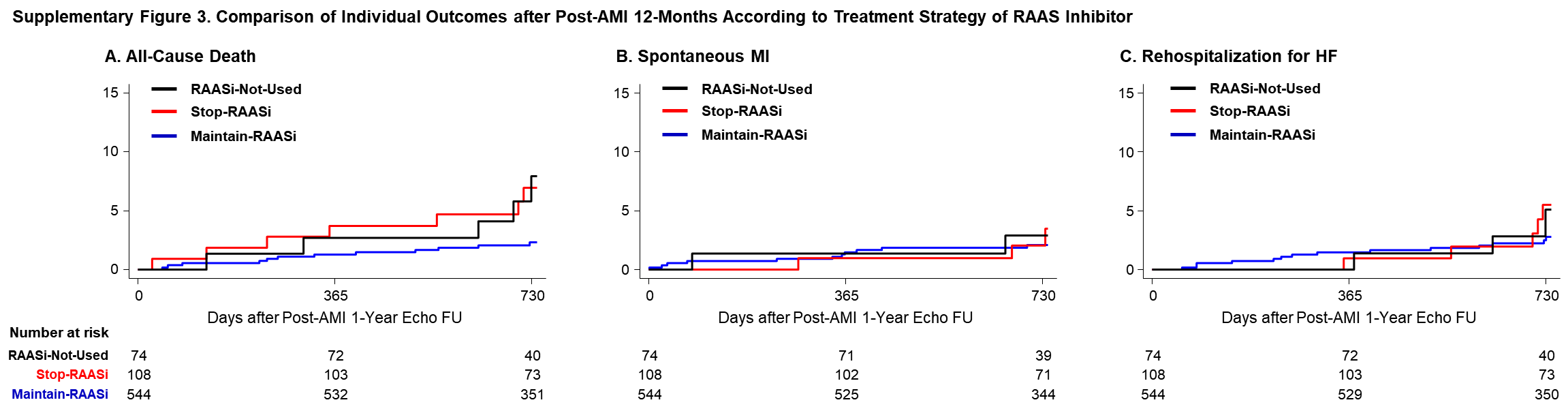


Comparison of cumulative incidence and Kaplan-Meier curves of (A) all-cause death, (B) spontaneous MI, and (C) rehospitalization for HF among the 3 groups; Maintain-RAASi, Stop-RAASi, and RAASi-Not-Used groups, are presented.

Abbreviations: AMI, acute myocardial infarction; HF, heart failure; MI, myocardial infarction; RAASi, Renin-Angiotensin-Aldosterone System inhibitor.
